# Elevated ALT correlates with severe liver injury, monocyte infiltration, and Pro-inflammatory immune dysregulation in chronic hepatitis B virus infection

**DOI:** 10.3389/fmed.2026.1865627

**Published:** 2026-09-04

**Authors:** Jian Huang, Ning Xu, Xiuyan Liang, Defeng Cai

**Affiliations:** 1 Department of Clinical Laboratory (Pathology) Centre, Medical School, South China Hospital, Shenzhen University, Shenzhen, China; 2 Guangdong Medical University, Dongguan, China; 3 Institute of Biopharmaceutical and Health Engineering, Tsinghua Shenzhen International Graduate School, Tsinghua University, Shenzhen, China

**Keywords:** ALT, HBV infection, hepatic immune dysregulation, liver injury, monocyte, wnt/*β*-catenin signaling pathway

## Abstract

**Background:**

Hepatitis B virus (HBV) infection remains a global health burden. Although alanine aminotransferase (ALT) is a well-established marker of liver injury, its relationship with immune infiltration, monocyte activation, and inflammatory immune skewing in chronic HBV infection remains incompletely understood. This study aimed to investigate the associations of ALT with liver injury, monocyte infiltration, and hepatic immune microenvironment dysregulation.

**Methods:**

A total of 192 HBV-infected patients and 37 healthy controls were enrolled. Serum ALT, AST, HBV seromarkers, HBV-DNA, alpha-fetoprotein (AFP), and serum fibrosis markers (COLIV, HA, PIIINP, LN) were measured. Univariate and multivariate regression analyses were used to adjust for confounders including age, sex, HBV-DNA, fibrosis markers, and HBeAg status. Transcriptomic data from the GSE84044 liver biopsy cohort (*n* = 105) were analyzed using the xCell algorithm to evaluate hepatic immune infiltration, macrophage polarization, and immune scores. Gene set enrichment analysis (GSEA) was performed to explore monocyte chemotaxis, activation, and WNT/*β*-catenin signaling pathway.

**Results:**

Chronic hepatitis B (CHB) patients showed significantly elevated ALT and AST. Higher ALT levels were strongly associated with elevated fibrosis markers and AFP, indicating more severe liver injury. Peripheral monocytes count and intrahepatic monocyte infiltration were both significantly increased in high-ALT patients. However, after multivariate adjustment, ALT was no longer independently associated with monocyte infiltration, suggesting that the association was largely attributable to overall disease severity rather than a specific ALT-related association. GSEA showed that ALT was positively correlated with monocyte chemotaxis, activation, macrophage differentiation, and WNT/*β*-catenin signaling pathway. Immune landscape analysis revealed a pro-inflammatory immune profile in high-ALT patients, characterized by increased M1 macrophages, CD8+ T cells, and elevated immune scores, accompanied by decreased Tregs and Th1 cells.

**Conclusion:**

Elevated ALT in chronic HBV infection correlates with severe liver injury and a pro-inflammatory, dysregulated hepatic immune microenvironment characterized by enhanced monocyte infiltration and M1 macrophage polarization. Transcriptomic associations reveal a statistical link between WNT/*β*-catenin signaling activation and hepatic immune dysregulation in HBV-related liver injury; these correlative findings provide preliminary clues for developing immunomodulatory therapeutic strategies for CHB.

## 1 Introduction

Hepatitis B virus (HBV) infection remains a significant global health challenge, affecting millions of individuals worldwide and driving substantial liver-related morbidity and mortality (1–3). Chronic HBV infection triggers progressive hepatic damage, including fibrosis, cirrhosis, and hepatocellular carcinoma (HCC), with the severity of liver damage often closely associated with alanine aminotransferase (ALT) (4–6). As the gold-standard biomarker for necroinflammation, ALT is routinely applied to monitor disease activity and guide antiviral treatment initiation among CHB patients (7). Nevertheless, the associations between ALT levels, liver damage, and immune response in HBV infection remain poorly defined. Recent cohort studies have demonstrated that serum ALT alone cannot reliably discriminate mild necroinflammation from severe intrahepatic monocyte infiltration (8, 9). Clinical and histopathological studies further indicate that hepatic CD68 + macrophage density and peripheral CD16 + pro-inflammatory monocyte frequencies correlate positively with serum ALT levels (10, 11). Transcriptomic profiling of liver biopsies has identified distinct intrahepatic immune microenvironments, with longitudinal data showing that ALT normalization parallels downregulation of hepatic immune infiltrates (12). Complementing these observations, imaging mass cytometry has revealed spatial associations between hepatic immune cell densities and serum ALT elevations across disease phases (13). Emerging single-cell transcriptomic evidence suggests that infiltrating hepatic monocytes and M1-polarized macrophages are associated with hepatocyte necrosis, yet few clinical studies systematically characterized how graded ALT elevation correlates with alterations in the comprehensive hepatic immune microenvironment, including adaptive T cell subsets and regulatory immune populations (14, 15). Very recent longitudinal single-cell analyses have identified a dynamic inflammatory monocyte-derived macrophage population that emerges during ALT elevation and regresses upon treatment-induced ALT normalization (16), underscoring the dynamic myeloid-ALT relationship. In addition, preclinical models have shown that WNT/*β*-catenin signaling is associated with monocyte chemotaxis and upregulation of CCR2, but clinical evidence examining the associations among ALT levels, WNT/*β*-catenin signaling activation, and intrahepatic monocyte recruitment in human CHB liver tissues is currently lacking (17).

Host immunity governs the entire pathogenic trajectory of HBV infection. While the immune system is essential for viral clearance, an exaggerated, unbalanced immune activation aggravates hepatocellular destruction and accelerates fibrotic progression (18, 19). Monocytes and macrophages constitute the core innate immune compartment infiltrating the liver during chronic HBV infection, where they orchestrate local inflammatory cascades and extracellular matrix deposition (20).

The hepatic immune microenvironment in CHB undergoes dynamic alteration across disease phases, transitioning from immune tolerance—characterized by quiescent effector responses and intact regulatory T-cell function—to immune clearance, marked by enhanced cytotoxic T-cell activity, diminished regulatory control, and pro-inflammatory macrophage polarization (19, 21, 22). However, the quantitative relationship between this immune skewing and graded ALT elevation remains poorly defined. Even so, the specific associations of these immune cells with elevated ALT and liver damage have not been fully characterized. Additionally, the WNT/*β*-catenin signaling, which is involved in various cellular processes, including cell proliferation, differentiation, and immune regulation, has been associated with liver disease progression (23–25). Its potential association with monocyte chemotaxis and activation during HBV infection requires comprehensive clinical validation. Despite abundant preclinical observations, human clinical data connecting ALT, myeloid cell infiltration, and WNT/*β*-catenin signaling cascade activation remain scarce. Notably, ALT elevation may reflect overall hepatic inflammatory burden rather than exerting independent immunomodulatory effects *per se*. Whether the association between ALT and monocyte infiltration persists after adjustment for confounding markers of disease severity—including fibrosis stage, viral replication, and HBeAg status—remains unresolved.

Collectively, existing literature has separately described the independent roles of ALT, monocyte-macrophage populations, and WNT/*β*-catenin signaling, and immune dysregulation in CHB progression, yet four critical research gaps remain unaddressed: First, at the clinical validation level, no dual validation combining large-scale clinical serum cohorts and paired liver biopsy transcriptomic datasets has been performed to dissect both systemic and intrahepatic correlations between ALT and monocyte infiltration. Second, at the immune atlas level, no full xCell-generated immune atlas covering 64 stromal and immune cell subsets has been constructed to quantify pro-inflammatory immune skewing across distinct ALT strata. Third, at the mechanistic level, human liver transcriptomic evidence characterizing the associations among ALT elevation, and WNT/*β*-catenin signaling cascade activation, and monocyte recruitment remains insufficient. Fourth, at the inferential level, it remains unclear whether ALT is independently associated with monocyte accumulation after adjustment for major confounders, including viral load, serum fibrosis markers, and HBeAg status. To resolve these gaps, the present study integrated a single-center clinical cohort of 192 CHB patients and the public liver biopsy transcriptomic dataset GSE84044 (*n* = 105). We systematically quantified the associations between ALT levels and multiple disease-related clinical endpoints: overall liver damage severity, circulating and intrahepatic monocyte enrichment, disordered pro-inflammatory immune microenvironment, and transcriptional activation of the WNT/*β*-catenin signaling. Our integrated clinical and transcriptomic analyses aim to identify statistical associations that may generate hypotheses for novel immunomodulatory therapies targeting monocyte recruitment and macrophage polarization for chronic HBV infection. Characterizing the ALT-associated immune dysregulation patterns may generate hypotheses for patient stratification beyond conventional virological markers and for CCR2- or WNT/*β*-catenin-targeted interventions to mitigate necroinflammation and fibrotic progression.

## 2 Materials and methods

### 2.1 Patients

One hundred and ninety-two patients with HBV who received care at the South China Hospital of Shenzhen University, Shenzhen between September, 2023, and April, 2024 were included in this study (Supplementary Table 1). The inclusion criteria were as follows: (1) Positive for hepatitis B surface antigen (HBsAg), (2) having undergone HBV-DNA testing. The exclusion criteria were: (1) Malignancy, (2) Hematological diseases, (3) a history of other chronic liver diseases (e.g., infection with hepatitis C virus and autoimmune hepatitis), (4) the presence of other serious medical conditions, and (5) missing data. In addition, thirty-seven healthy controls who underwent physical examination in South China Hospital of Shenzhen University were selected as the control group (Supplementary Table 2). Data on antiviral treatment status [e.g., nucleos(t)ide analogues or interferon], HBV disease phase (immune tolerant, immune active, inactive carrier), and liver fibrosis staging were not systematically collected in this retrospective cohort and were therefore unavailable for analysis.

### 2.2 Public dataset (GSE84044)

To validate the findings from our clinical cohort and investigate immune infiltration patterns, we utilized the publicly available GSE84044 dataset from the Gene Expression Omnibus (GEO) database (https://www.ncbi.nlm.nih.gov/geo/). This dataset comprises 124 liver biopsy samples from chronic hepatitis B (CHB) patients who underwent diagnostic liver biopsy at Ruijin Hospital, Shanghai Jiaotong University School of Medicine. The GSE84044 cohort included treatment-naïve CHB patients with available clinical and virological data. Liver biopsies were performed for clinical staging purposes, and RNA was extracted from fresh-frozen liver tissue. Clinical metadata available for these patients included age, sex, ALT levels, AST levels, HBeAg status, and HBV-DNA levels. Patients in the GSE84044 cohort were stratified into high-ALT and low-ALT subgroups using serum ALT levels as the stratification parameter. Specifically, patients were first separated by biological sex and then classified into the high-ALT subgroup when their serum ALT exceeded the sex-specific threshold (male >30 IU/L, female >19 IU/L), otherwise assigned to the low-ALT subgroup. These cut-offs were uniformly defined according to the 2022 Chinese expert consensus on expanded antiviral therapy for chronic hepatitis B and the 2016 AASLD guidelines. Participants with available ALT data were first stratified by sex; those exceeding the sex-specific threshold were assigned to the high-ALT subgroup, and all others to the low-ALT subgroup. Patients with missing ALT values or incomplete sex information were excluded before stratification, yielding 105 patients for downstream analysis (Supplementary Table 3).

### 2.3 Observation indicators

We collected laboratory data including patient age, sex, complete blood count, liver and renal biochemistry, HBV-DNA viral load, and serum alpha-fetoprotein (AFP). Peripheral blood routine testing was conducted on a Mindray BC-7500 automated hematology analyzer (Mindray, Shenzhen, China). The HBV-DNA level was measured using the ABI QuantStudio Dx real-time PCR instrument and the HBV-DNA detection kit (DAAN Gene, China), with a detection limit of 30 IU/mL. The liver and kidney function indicators, AFP, HBsAg, HBsAb, HBeAg, HBeAb, HBcAb, and serum fibrosis markers were detected by biochemical immunoassay (model AU5800, Beckman Coulter, Brea, California, USA). Hepatic fibrosis was assessed using four serum biomarkers: Type IV Collagen (COLIV), Hyaluronic Acid (HA), Procollagen III N-terminal Peptide (PIIINP), and Laminin (LN). We acknowledge that these are indirect, surrogate markers of fibrosis that may be influenced by necroinflammatory activity and do not represent direct histological assessment. Liver biopsy with METAVIR staging or transient elastography (FibroScan) was not performed in this cohort. Patient characteristics and laboratory results are shown in Supplementary Table 1, and data from healthy controls are presented in Supplementary Table 2.

### 2.4 Immune microenvironment analysis

To investigate hepatic immune infiltration patterns associated with ALT elevation, we applied the xCell algorithm to RNA-seq data from the GSE84044 liver biopsy cohort. xCell is a robust algorithm designed to analyze the infiltration levels of 64 immune and stromal cell types, encompassing extracellular matrix cells, epithelial cells, hematopoietic progenitors, as well as innate and adaptive immune cells (26, 27). This algorithm was utilized to assess the infiltration of lymphocytes and myeloid cells between two subgroups. The xCell algorithm was used to compute microenvironment scores, stromal, and immune scores.

### 2.5 Gene Set enrichment analysis

To validate the findings obtained from pathway enrichment analysis, we performed gene set enrichment analysis (GSEA) (28) using RNA-seq data from liver biopsies in the GSE84044 cohort. This tissue-based transcriptomic analysis complemented the serum biomarker data from our clinical cohort. GSEA is a computational method used to determine whether predefined gene sets exhibit statistically significant, coordinated differences between two biological states, such as different phenotypes. The key parameters for our analysis were set as follows: we performed 1,000 permutations, used the weighted enrichment statistic and applied the Signal2Noise metric for gene ranking. The maximum gene set size was set to 500, and the minimum to 15. The gene sets of monocyte chemotaxis, monocyte activation pathways, and macrophage differentiation were obtained from the Molecular Signatures Database (MSigDB). The selection criteria were a False Discovery Rate (FDR) of less than 0.05 and an absolute Normalized Enrichment Score (NES) greater than 1. For heatmap visualization (Figure 6E), the top 17 WNT/*β*-catenin pathway genes ranked by absolute Spearman correlation coefficient (|*ρ*|) with serum ALT were selected as representative genes to improve clarity. GSEA (Figures 6A–C, F) was performed on the complete, unfiltered gene set without pre-screening for differential expression. For gene-ALT association analyses, Spearman's rank correlation was employed, given the heavily right-skewed distribution of liver tissue RNA-seq data, ensuring robustness against outliers and distributional violations. All GSEA was performed on RNA-seq data from the GSE84044 liver biopsy cohort using the parameters defined above.

### 2.6 Statistical analysis

#### 2.6.1 Study design and general statistical framework

This study employed a cross-sectional design with independent, unmatched groups (no paired or repeated within-subject measurements). All statistical analyses were performed using SPSS 26.0 and R 4.0.5 (http://www.R-project.org) with complementary R packages. For two-group comparisons, the independent sample t-test was adopted for normally distributed data with homogeneous variance; for skewed non-normal data, the Wilcoxon rank-sum test (Mann–Whitney U test) was used, consistent with our independent, unmatched design. The Wilcoxon signed-rank test was not applicable, as it requires paired or repeated measurements from the same subject (e.g., pre-treatment vs. post-treatment), whereas our cohort contained no within-subject repeated sampling.

#### 2.6.2 Regression analysis

Univariate and multivariate linear regression models were built to identify independent predictors of peripheral monocyte count. Covariates included ALT, age, sex, HBV-DNA, serum fibrosis markers (COLIV, HA, PIIINP, LN), AFP, and HBeAg status. Multicollinearity was assessed by variance inflation factor (VIF), with VIF < 5 indicating acceptable collinearity. Variables with FDR-adjusted q < 0.10 in univariate screening were entered into multivariate models.

#### 2.6.3 Correlation analysis

Given the strong skewness of serum biochemical and liver transcriptomic data, which violates the normality prerequisite of parametric Pearson correlation, non-parametric Spearman's rank correlation (*ρ*) was used for all gene-ALT association analyses. Correlation strength was interpreted as: |*ρ*| 0.00–0.19 (very weak), 0.20–0.39 (weak), 0.40–0.59 (moderate), 0.60–0.79 (strong), and 0.80–1.0 (very strong) (29).

#### 2.6.4 Multiple testing correction strategy

FDR correction was applied selectively based on the nature of the analysis. For single biomarker comparisons between two independent groups—such as ALT or AST levels between healthy controls and CHB patients, or COLIV, HA, PIIINP, and LN between high-ALT and low-ALT subgroups—raw two-sided *P* values from Wilcoxon rank-sum tests are reported without multiple testing correction, as each comparison tests a distinct, pre-specified biological hypothesis independently. In contrast, Benjamini–Hochberg FDR correction was mandatory for four categories of parallel analyses where multiple related endpoints were tested simultaneously: (i) xCell immune/stromal cell deconvolution covering 64 subsets; (ii) gene set enrichment analysis (GSEA) across multiple gene sets; (iii) Spearman correlation across 17 WNT/*β*-catenin pathway genes; and (iv) univariate regression screening of 8 candidate predictors. FDR-adjusted q < 0.05 was applied as the uniform significance cutoff for all corrected analyses, except for univariate regression screening where q < 0.10 was used as the entry criterion for multivariate modeling. Adjusted q-values are displayed in all corresponding tables and figures.

## 3 Results

### 3.1 CHB patients with higher ALT exhibit relatively severe liver injury

**Figure 1:**
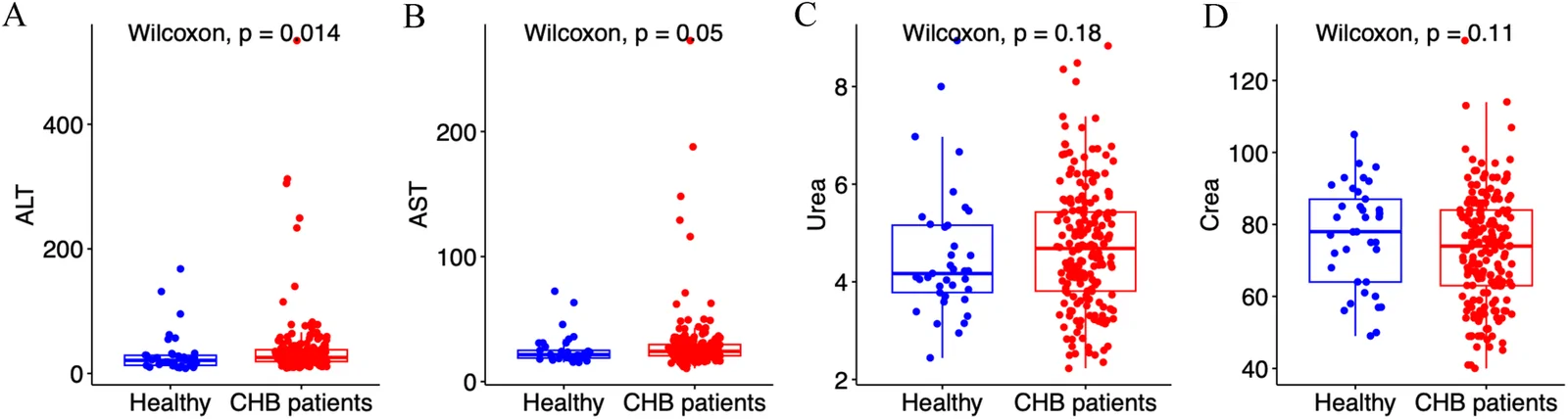
CHB patients exhibited liver injury. Comparative analysis of Liver and Kidney function biomarkers: AST, ALT, Urea and Crea in CHB patients vs. healthy controls **(A–D)**. Raw two-sided Wilcoxon rank-sum *P* values are reported for independent single-biomarker comparisons **(A–D)** without multiple testing correction, as each panel evaluates a distinct biological hypothesis. No FDR adjustment was applied.

To investigate the clinical characteristics of HBV infection, we compared liver and kidney function indicators between CHB patients and healthy controls. We observed significantly elevated ALT and AST levels in the serum of CHB patients compared to healthy controls (Figures 1A,B). However, there was no significant difference in the indicators of kidney function (Figures 1C,D). These results are consistent with previous studies, which have shown that HBV infection primarily affects the function of the liver.

**Figure 2:**
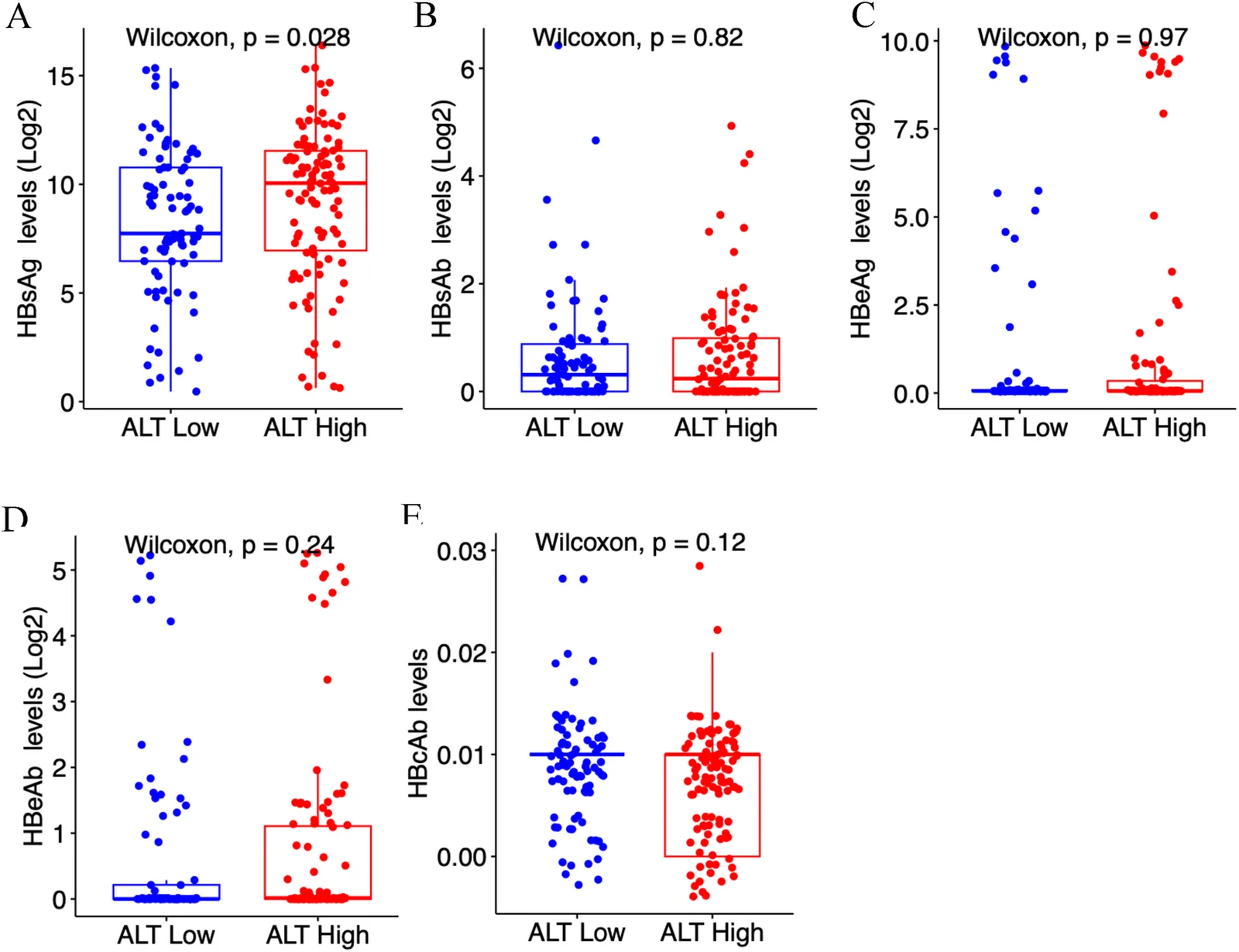
CHB patients with higher ALT show more HBsAg. HBV serological profile (HBsAg, HBsAb, HBeAg, HBeAb, HBcAb) in ALT high vs. low CHB patients **(A–E)**. Raw two-sided Wilcoxon rank-sum *P* values are reported for independent single-biomarker comparisons **(A–E)** without multiple testing correction. Each serological marker was tested independently against ALT strata; no FDR adjustment was applied.

ALT is often used as a biomarker for liver damage, and its levels can reflect the extent of hepatocellular damage (30). Internationally, multiple guidelines have set the treatment threshold for ALT at 30 IU/L for men and 19 IU/L for women (31–33). We found that patients with high levels of ALT (>19 IU/L for females and >30 IU/L for males) in their serum also exhibited higher levels of HBsAg (Figure 2A). However, no significant differences were observed in other hepatitis B markers: HBsAb, HBeAg, HBeAb, and HBcAb (Figures 2B–E).

**Figure 3:**
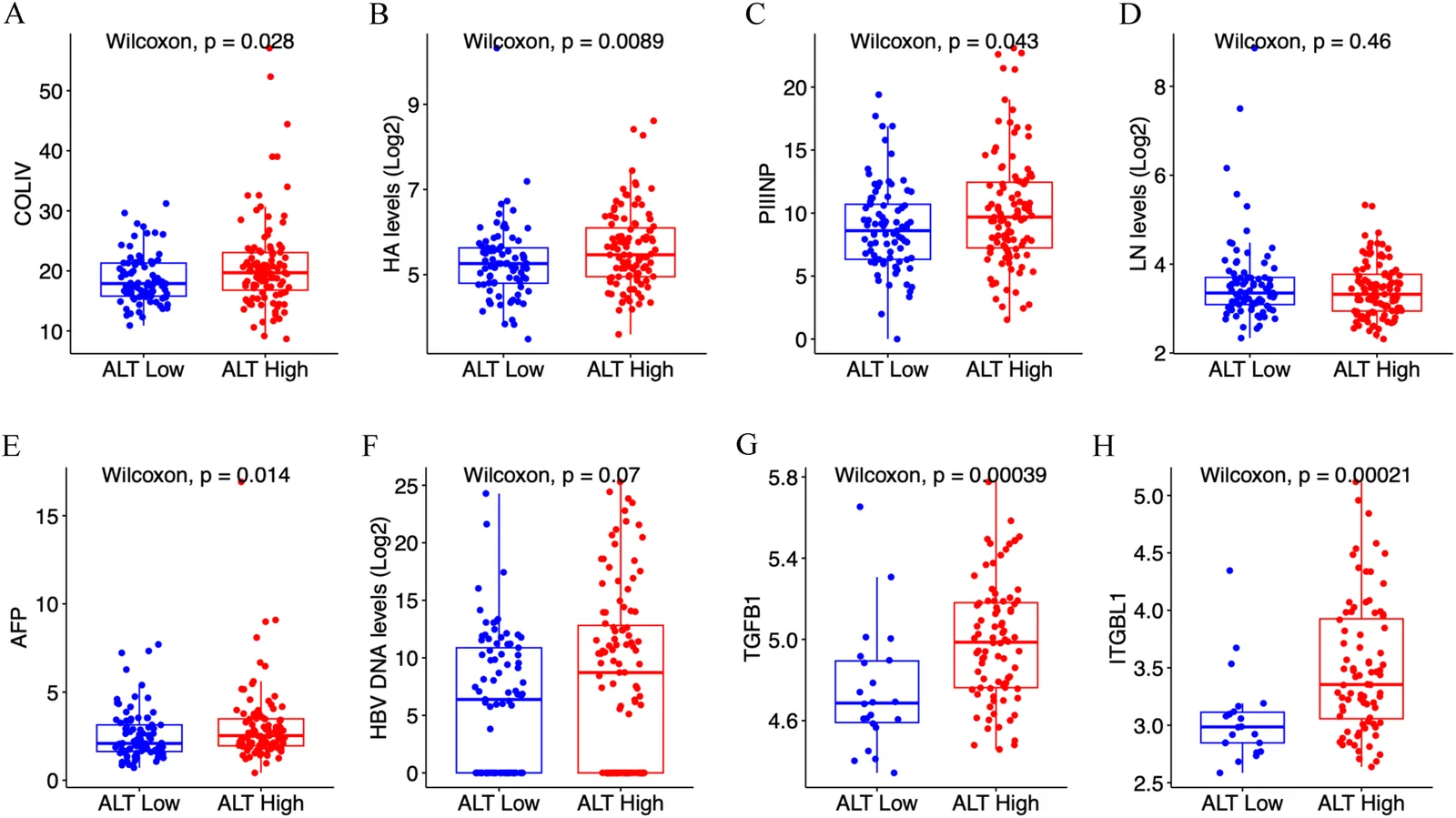
CHB patients with elevated ALT exhibit elevated serum markers of extracellular matrix turnover. **(A–D)** Serum levels of surrogate fibrosis markers: **(A)** Collagen Type IV (COLIV), **(B)** Hyaluronic Acid (HA), **(C)** Procollagen III N-terminal Peptide (PIIINP), and **(D)** Laminin (LN). **(E)** Serum Alpha-fetoprotein (AFP) and **(F)** HBV-DNA levels. (log2 IU/mL). **(G,H)** Expression of fibrogenesis regulatory factors TGF-*β*1 **(G)** and ITGBL1**(H)** from GSE84044 cohort. For serum biomarker comparisons **(A–F)**, raw two-sided Wilcoxon rank-sum *P* values are reported without multiple testing correction, as each marker represents an independent biological endpoint. For hepatic gene expression comparisons **(G,H)**, Benjamini–Hochberg FDR correction was applied across the two parallel tests (TGF-*β*1 and ITGBL1), with FDR-adjusted q < 0.05 as the significance threshold.

While ALT can be a useful indicator of liver health in CHB patients, it does not always accurately reflect the severity of liver damage or the necessity for treatment. Using serum surrogate markers of fibrosis, we observed that patients with high levels of ALT had elevated COLIV, HA, and PIIINP compared to those with low levels of ALT, whereas LN was not significantly elevated (Figures 3A–D), and they also exhibited higher AFP in serum (Figure 3E). Additionally, although there was no statistically significant difference in HBV-DNA, a tendency towards active replication was observed in patients with higher ALT (Figure 3F). Previous studies have identified ITGBL1 and TGF-*β*1 as factors associated with fibrogenesis, with ITGBL1 being associated with liver fibrosis in relation to TGF-*β*1 signaling (34). In the GSE84044 cohort, both TGF-*β*1 and ITGBL1 mRNA transcripts were significantly upregulated in the high-ALT subgroup (Figures 3G,H, FDR-adjusted q < 0.05), suggesting coordinated activation of profibrogenic signaling pathways in the hepatic microenvironment of patients with elevated ALT.

### 3.2 CHB patients with higher ALT show elevated monocyte count

**Figure 4:**
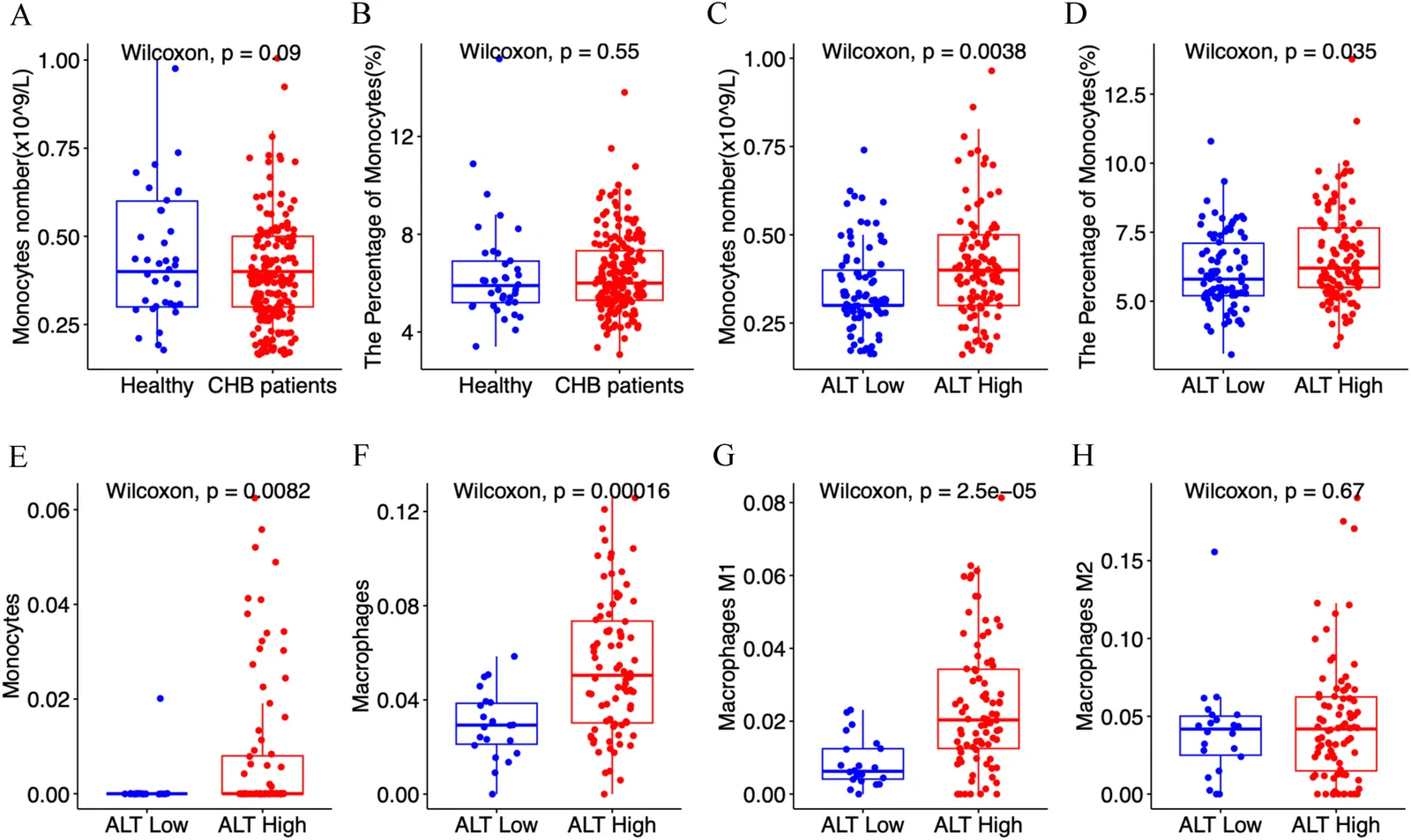
CHB patients with elevated ALT exhibit increased monocytes and macrophage activation. **(A–D)** Clinical cohort data: Peripheral blood monocyte count **(A)** and percentage **(B)** in CHB patients vs. healthy controls. Monocyte count **(C)** and percentage **(D)** in CHB patients with elevated ALT vs. normal ALT. **(E–H)** GSE84044 validation cohort: xCell algorithm-based enrichment scores for **(E)** monocytes, **(F)** total macrophages, **(G)** M1, and **(H)** M2 macrophages in liver transcriptomes. For peripheral blood biomarker comparisons **(A–D)**, raw two-sided Wilcoxon rank-sum *P* values are reported without multiple testing correction. For hepatic immune infiltration analysis **(E–H)**, Benjamini–Hochberg FDR correction was applied across all 64 immune and stromal cell subsets deconvoluted by xCell, with FDR-adjusted q < 0.05 as the significance threshold.

The clinical outcome of HBV infection depends on the complex interplay between viral replication and the host's immune response. Innate immunity plays an important role in the early stages of disease (20, 35). We observed no statistically significant differences in absolute monocyte count or monocyte percentage between CHB patients and healthy controls (Figures 4A,B). In contrast, when stratified by ALT levels, an increase in monocyte count was observed in patients with higher ALT (Figures 4C,D). Given that elevated ALT may reflect overall disease severity rather than a specific independent association, we performed additional analyses to assess whether this association was independent of fibrosis stage, viral replication, and other clinical parameters. Univariate analysis revealed that ALT (*β* = 0.198, *P* = 0.006), sex (male vs. female, *β* = 0.276, *P* < 0.001), and HBV-DNA levels (negative vs. positive, *β* = 0.198, *P* = 0.006) were significantly associated with monocyte count. Notably, in multivariate linear regression analysis, after adjusting for sex and HBV-DNA levels (the only covariates meeting the FDR-adjusted q < 0.10 entry criterion in univariate screening), ALT was no longer independently associated with monocyte count (*β* = 0.128, *P* = 0.067). Fibrosis markers and HBeAg status were screened but did not meet the entry criterion and were therefore not retained in the final model, suggesting that the univariate association was largely attenuated and confounded by markers of overall disease severity. While this *P* value does not reach conventional statistical significance, its proximity to the 0.05 threshold indicates a residual trend that may warrant validation in larger cohorts (Table1).

To validate the association between ALT elevation and monocyte infiltration observed in our clinical cohort, we analyzed the GSE84044 liver transcriptome dataset. While our clinical data showed elevated peripheral blood monocyte count in high-ALT patients (Figures 4C,D), the GSE84044 dataset enabled a direct assessment of intrahepatic immune infiltration. In the GSE84044 cohort, hepatic monocyte infiltration was significantly enriched in the high-ALT group (Figure 4E, FDR-adjusted q < 0.05 across all 64 immune/stromal cell signatures). Consistent with the established association of monocytes with tissue macrophages, xCell analysis further revealed that livers from high-ALT patients exhibited a higher proportion of total macrophages and, specifically, pro-inflammatory M1 macrophages (Figures 4F–H, FDR-adjusted q < 0.05). This aligns with previous studies indicating that monocytes recruited to sites of inflammation can differentiate into either pro-inflammatory M1 or anti-inflammatory M2 macrophages, depending on the local microenvironment (21, 36).

**Figure 5:**
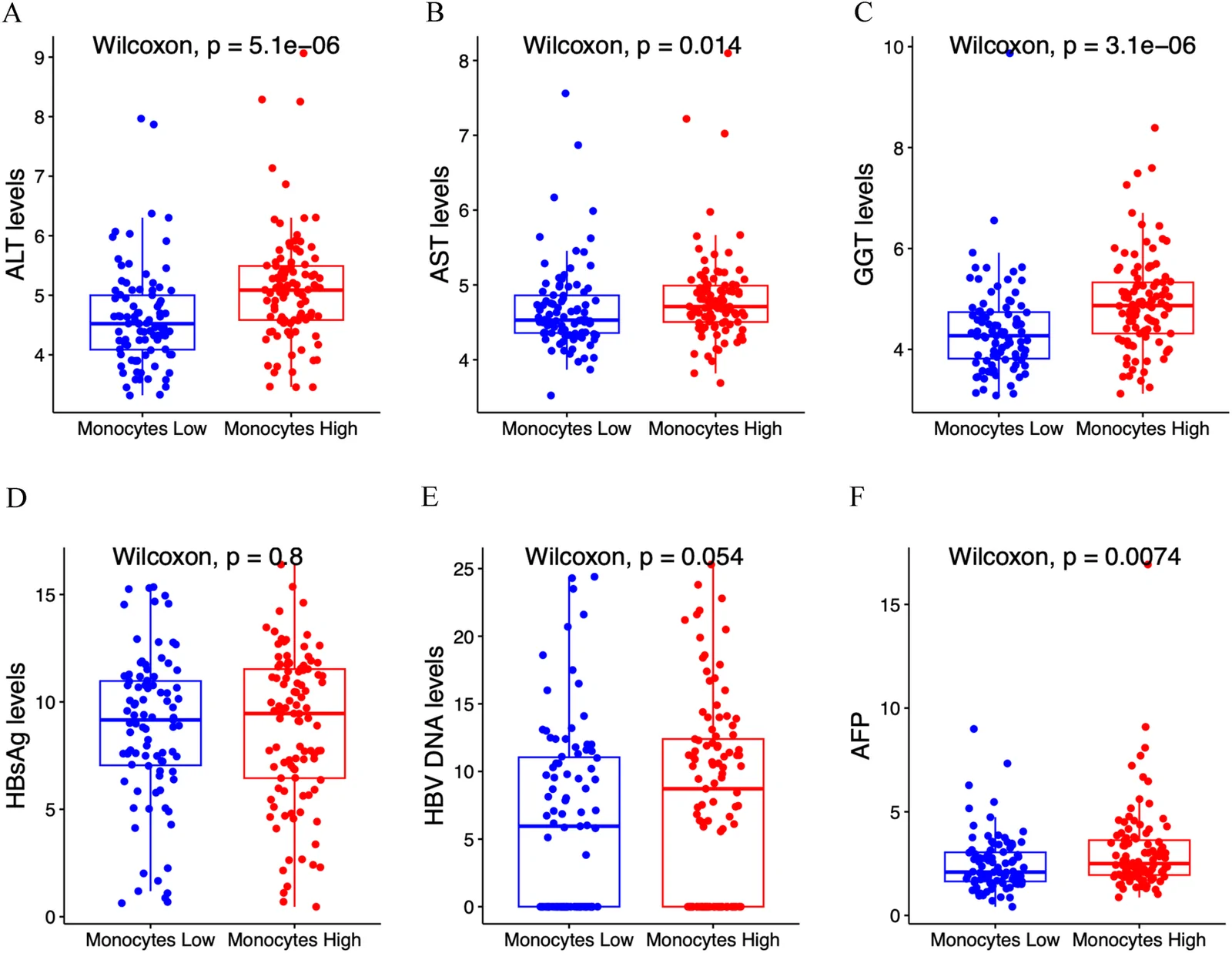
Monocytes are associated with liver injury in CHB patients. **(A–C)** Serum levels of **(A)** ALT, **(B)** AST, and **(C)** GGT in patients with high vs. low monocyte count. **(D,E)** Viral biomarkers: **(D)** HBsAg and **(E)** HBV-DNA levels. **(F)** Serum AFP levels. Raw two-sided Wilcoxon rank-sum *P* values are reported for independent single-biomarker comparisons **(A–F)** without multiple testing correction. Each biomarker was evaluated separately against monocyte count strata; no FDR adjustment was applied.

Monocytes play a significant role in the liver, particularly in the context of liver diseases such as hepatitis and cirrhosis (37, 38). Our results show that patients with greater monocyte infiltration had more severe liver damage (Figures 5A–C). We also observed that higher monocytes were associated with increased AFP and active HBV replication, but these did not reflect changes in HBsAg (Figures 5D–F). Collectively, these findings indicate that higher monocyte counts identify a subset of CHB patients with more severe liver damage and active viral replication. Integrating these clinical data with the hepatic transcriptomic findings from GSE84044 (Figures 4E–H), ALT elevation is associated with monocyte-mediated inflammatory infiltration and M1 macrophage polarization in the liver, though this association is attenuated after adjustment for overall disease severity (Table 1).

**Table 1:** Univariate and multivariate linear regression analyses of factors associated with monocyte count. .

Variable	Univariate Analysis			Multivariate Analysis		
	B (95% CI)	*β*	*P*-value	B (95% CI)	β	*P*-value
ALT	0.001 (0.000–0.001)	0.198	0.006	<0.001 (0.000–0.001)	0.128	0.067
Age	−0.002(−0.004–0.001)	−0.098	0.176			
Gender (male VS female)	0.082 (0.041–0.123)	0.276	<0.001	0.078 (0.038–0.119)	0.256	<0.001
HBV-DNA (negative vs. positive)	0.058 (0.017–0.099)	0.198	0.006	0.058 (0.019–0.097)	0.199	0.004
COLIV	−0.001(−0.004–0.002)	−0.057	0.429			
PIIIPNP	−0.001(−0.006–0.004)	−0.032	0.655			
LN	<0.001(−0.001–0.001)	0.004	0.951			
HA	<0.001(−0.000–0.000)	−0.023	0.752			
HBeAg (negative vs. positive)	−0.005 (0.061–0.05)	−0.015	0.837			

CI, confidence interval.

### 3.3 Coordinated activation of WNT/*β*-catenin signaling pathway and monocyte infiltration in patients with elevated ALT

**Figure 6:**
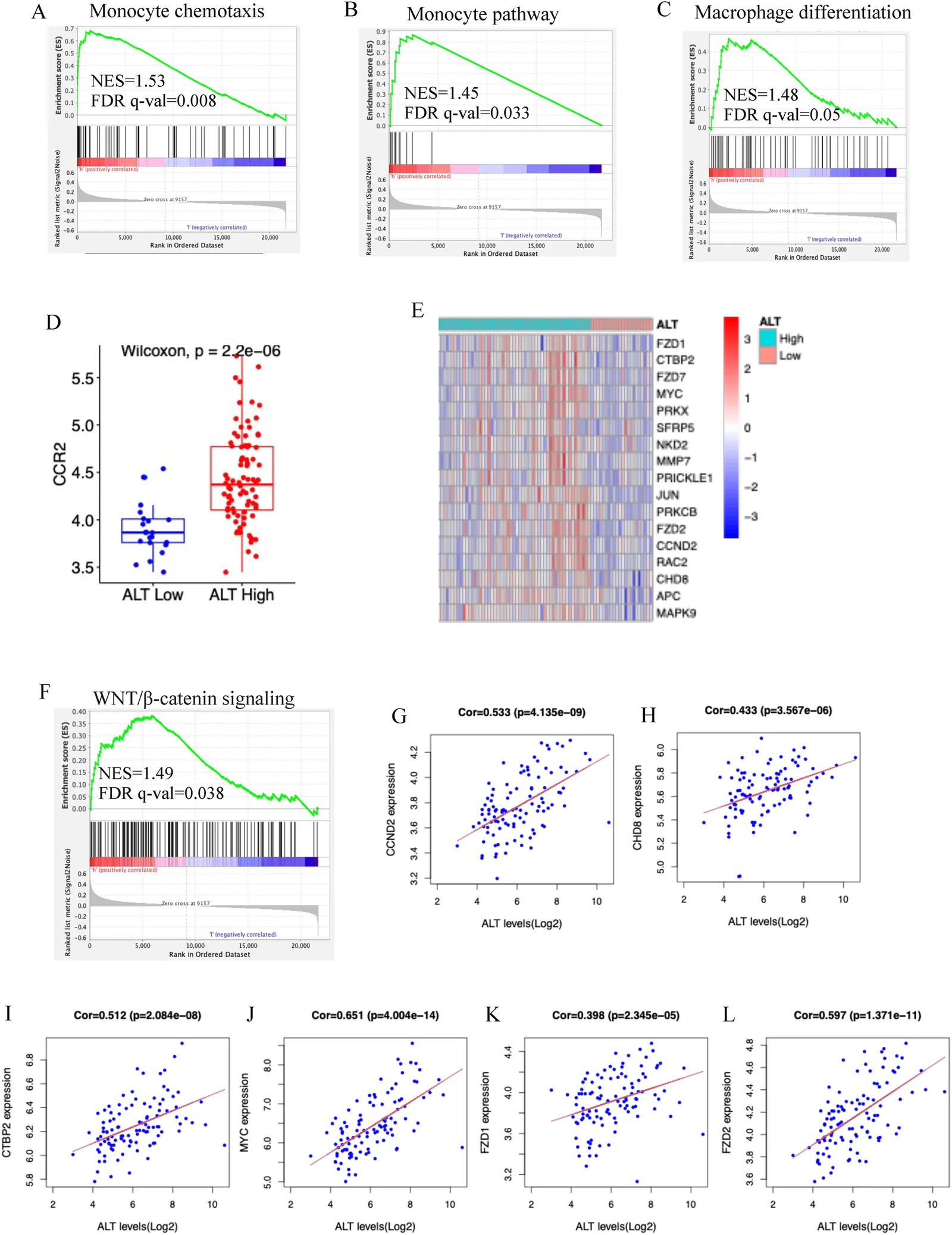
Association between ALT elevation, WNT/*β*-catenin signaling activation, and monocyte infiltration. **(A–C)** GSEA result showing positive association of ALT with **(A)** monocyte chemotaxis, **(B)** monocyte activation pathway, and **(C)** macrophage differentiation. **(D)** CCR2 mRNA expression is upregulated in high-ALT patients. **(E)** Heatmap of the top 17 WNT/*β*-catenin pathway genes ranked by absolute Spearman correlation (|*ρ*|) with serum ALT in patients stratified by ALT levels. **(F)** GSEA confirms enrichment of WNT/*β*-catenin signaling in higher ALT patients. **(G–L)** Spearman's rank correlation analysis of ALT with WNT/*β*-catenin pathway components. All statistical analyses in this figure followed unified criteria by analysis type. Benjamini–Hochberg FDR correction was applied for GSEA **(A–C,F)** across all tested gene sets and for Spearman's rank correlation **(G–L)** across 17 WNT/*β*-catenin pathway genes, with FDR-adjusted q < 0.05 defined as the significance cutoff. For single-gene two-group comparison **(D)**, raw two-sided Wilcoxon rank-sum *P* values are reported without multiple testing correction. Spearman's rank correlation (*ρ*) was utilized to assess associations between ALT and WNT/*β*-catenin pathway components; this non-parametric test was selected given the heavily skewed, non-normal distribution of liver transcriptomic and serum biochemical data, which violates the normality prerequisite of parametric Pearson correlation. For Spearman's rank correlation **(G–L)**, raw *p*-values are displayed for illustrative purposes; statistical significance was determined based on FDR-adjusted q < 0.05 across all 17 WNT/*β*-catenin pathway genes tested.

To further explore these molecular associations, we performed GSEA on GSE84044 liver RNA-seq datasets, followed by correlation testing between ALT and individual pathway gene transcripts. The results showed that ALT was positively associated with monocyte chemotaxis (Figure 6A), the monocyte activation pathway (Figure 6B), and macrophage differentiation (Figure 6C). The WNT/*β*-catenin signaling has been reported to be associated with CCR2 expression and monocyte accumulation in the liver (17, 39). CCR2 expression was significantly upregulated in CHB patients with higher ALT (Figure 6D). In addition, the findings also revealed that genes involved in the WNT/*β*-catenin pathway were enriched in patients with high levels of ALT, and ALT was positively correlated with the expression of these components (Figures 6E,F). Moreover, ALT was significantly correlated with APC, CCND2, CHD8, CTBP2, FZD1, FZD2, FZD7, JUN, MAPK9, MMP7, MYC, NKD2, PRICKLE1, PRKCB, PRKX RAC2 and SFRP5 via Spearman's rank correlation (FDR-adjusted q < 0.05, Figures 6G–L and Supplementary Figure 1) (40, 41), suggesting a potential linkage between ALT elevation, WNT/*β*-catenin signaling cascade activation, hepatic immune dysregulation, and systemic pro-inflammatory immune dysregulation. These correlative transcriptomic findings remain hypothesis-generating and require protein-level and functional experimental validation.

### 3.4 Immune landscape of elevated ALT in CHB patients reveals pro-inflammatory hepatic immune dysregulation

**Figure 7:**
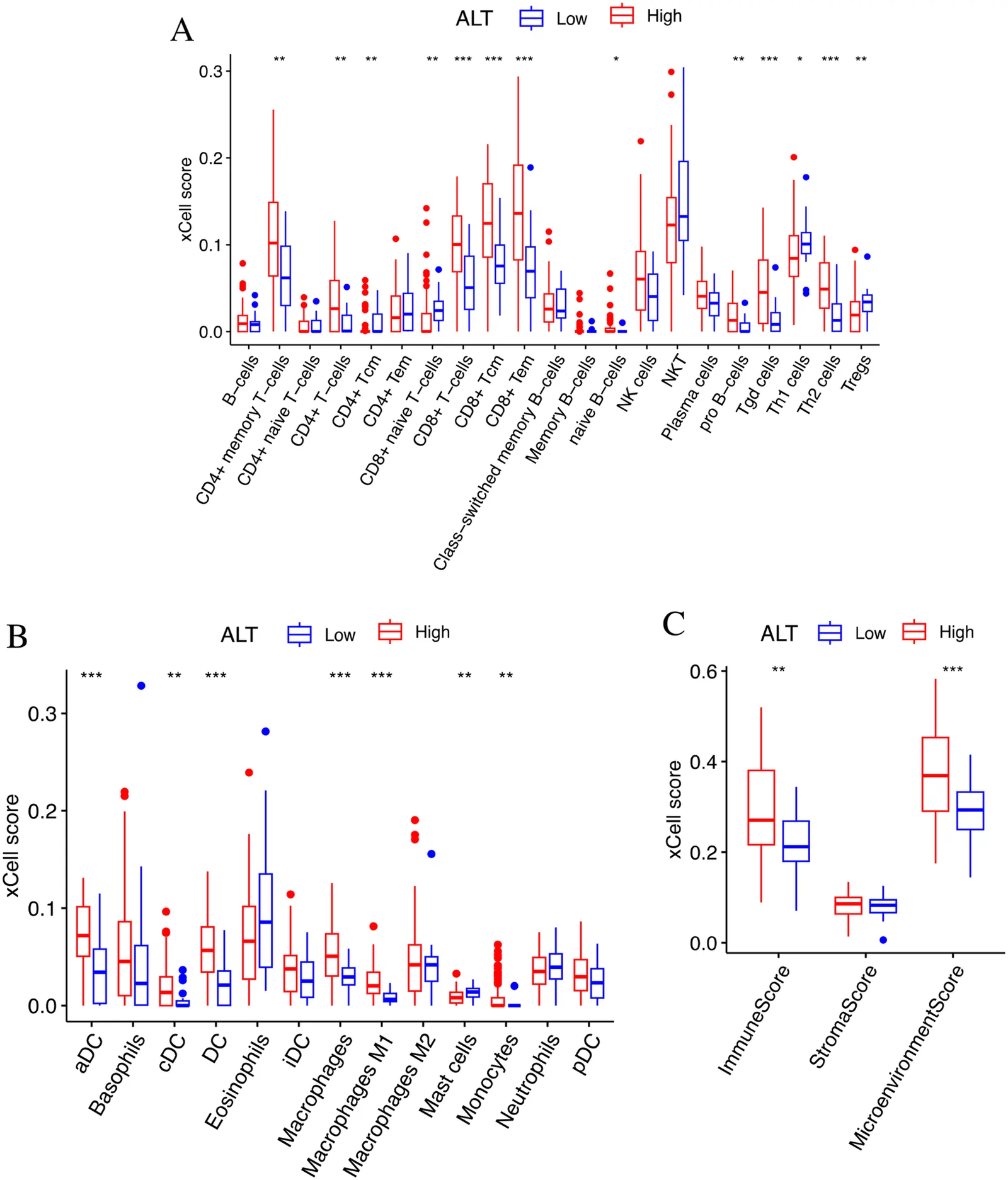
Immune landscape of ALT elevation in CHB patients (GSE84044 cohort). xCell analysis with FDR correction across 64 immune and stromal cell types. **(A,B)** The relationship between ALT and immune infiltration of **(A)** Lymphocytes and **(B)** Myeloid subsets using xCell. **(C)** The relationship between ALT and infiltrating fraction of Immune, stromal and Microenvironment score. Benjamini–Hochberg FDR correction was applied across all 64 immune and stromal cell subsets deconvoluted by xCell **(A,B)** and across the three microenvironment scores **(C)**, with FDR-adjusted q < 0.05 as the significance threshold. *FDR-adjusted q < 0.05; **FDR-adjusted q < 0.01; ***FDR-adjusted q < 0.001.

The innate immune response is the first line of defense against HBV, and plays a critical role in the recruitment and maturation of the adaptive immune response (42, 43). To further investigate the association between ALT and the immune microenvironment in CHB patients, we performed xCell analysis with FDR correction across all 64 cell types in GSE84044 dataset. The high-ALT group showed significantly altered immune composition, including increased proportions of CD4 + memory T cells, CD8+ T cells, M1 macrophages, and monocytes (FDR-adjusted q < 0.05), with significantly decreased Tregs and Th1 cells (FDR-adjusted q < 0.05) (Figures 7A,B). Notably, no significant increase in immunosuppressive cells (e.g., M2 macrophages) was observed in the higher ALT group. Contrary to a classical immunosuppressive phenotype, the immune profile in high-ALT patients was characterized by pro-inflammatory skewing: increased M1 macrophages and CD8+ T cells, decreased Th1 cells and Tregs, and elevated immune and microenvironment scores (Figures 7A–C). These results suggest that the immune microenvironment in CHB patients with higher ALT is characterized by pro-inflammatory immune activation and dysregulation.

## 4 Discussion

While the correlation between elevated ALT and hepatic necroinflammation is well established, this study advances the field through two contributions: the integration of a clinical cohort (*n* = 192) with the GSE84044 transcriptomic dataset (*n* = 105) for cross-compartment validation; and the first exploratory analysis linking ALT elevation to monocyte infiltration, M1 polarization, and WNT/*β*-catenin activation in CHB liver tissue. We found significantly increased monocyte infiltration in high-ALT patients, consistent with the established role of monocytes and macrophages in HBV-related liver inflammation and fibrosis (14, 21, 37, 44). These cells migrate to the liver and differentiate into macrophages that either drive inflammation or promote repair depending on the microenvironment (21, 36). In line with our inflammatory signature, Padilha et al. validated the interplay between circulating pro-inflammatory cytokines and persistent necroinflammation, reinforcing the link between myeloid-derived cytokine release and ALT flares (45).

The ALT–monocyte association was attenuated and lost statistical significance after multivariate adjustment for age, sex, HBV-DNA, fibrosis markers (COLIV, HA, PIIINP, LN), and HBeAg status (*β* = 0.128, *P* = 0.067; Table 1). This attenuation indicates that the univariate association was largely confounded by markers of overall disease severity, reinforcing that ALT functions as a composite biomarker of hepatic inflammatory burden rather than an independent driver of monocyte recruitment; thus, ALT should be interpreted as a composite marker of liver damage. Notably, transcriptomic analyses further linked activated WNT/*β*-catenin signaling—a pathway known for its roles in cell proliferation and immune regulation (23, 40)—to monocyte chemotaxis in CHB, with concomitant upregulation of CCR2, a chemokine receptor critical for monocyte recruitment, in higher-ALT patients (17). These correlative findings remain hypothesis-generating and require functional validation.

The immune profile observed in high-ALT patients requires careful interpretation. Classical immunosuppression is characterized by increased regulatory T cells (Tregs), M2 macrophages, and myeloid-derived suppressor cells, alongside decreased effector T cells and pro-inflammatory cytokines. In contrast, our data showed the opposite pattern: decreased Tregs and Th1 cells, alongside increased M1 macrophages and elevated CD8+ T cells. While Th1 reduction may seem counterintuitive in a pro-inflammatory context, it likely reflects HBV-specific adaptive immune exhaustion or compartmentalization rather than global immunosuppression. Collectively, this configuration—heightened innate effector responses coupled with diminished regulatory and antigen-specific adaptive control—reflects pro-inflammatory skewing rather than immunosuppression. This pattern aligns with the pathophysiology of chronic hepatitis B, where persistent viral antigen stimulation drives continuous immune activation, leading to necroinflammation and fibrosis (46). The elevated immune and microenvironment scores further support increased inflammatory infiltration rather than immune quiescence. Therefore, characterizing this profile as immunosuppressive would misrepresent the underlying biology; instead, the term dysregulation more appropriately captures this disordered state, with direct implications for therapeutic strategy. Specifically, immunosuppressive therapy would be contraindicated, whereas immunomodulatory approaches targeting specific inflammatory pathways may be warranted.

Clinically, our findings suggest that elevated ALT in CHB patients not only indicates hepatocellular injury but also marks dysregulated pro-inflammatory immune activation and increased monocyte infiltration. These insights could guide therapies targeting immune modulation, such as inhibiting monocyte recruitment or altering macrophage polarization. For instance, therapeutic strategies targeting the WNT/*β*-catenin signaling or the CCR2 axis represent a testable hypothesis to limit monocyte recruitment and excessive hepatic inflammation, which may theoretically slow fibrotic progression in CHB; dedicated functional studies are required to clarify whether these associations reflect causal mechanisms.

This study has several limitations. First, the retrospective, single-center design may introduce selection bias, as patients were recruited from a single tertiary hospital and may not fully represent the broader chronic hepatitis B population. Second, hepatic fibrosis was assessed using serum surrogate markers (COLIV, HA, PIIINP, and LN) rather than gold-standard histological METAVIR staging or transient elastography; these circulating matrix turnover products may be confounded by concurrent neuroinflammatory activity and lack uniform diagnostic cutoffs, potentially misclassifying fibrosis severity. Third, the GSE84044 cohort comprised treatment-naïve patients undergoing diagnostic liver biopsy, representing a potentially more advanced or selected population than our unselected clinical cohort. Differences in sample processing, measurement platforms, and clinical characteristics introduce technical heterogeneity that precludes direct quantitative comparison, although the consistent direction of associations across both cohorts supports biological validity. Fourth, systematic data on antiviral treatment status and standardized HBV disease phase (immune tolerant, immune active, inactive carrier) were unavailable, precluding stratified analyses. Finally, we acknowledge that residual selection bias and confounding by unmeasured covariates may remain despite multivariate adjustment and FDR correction.

## 5 Conclusion

Integrated analysis of our 192-subject clinical CHB cohort confirms that serum ALT elevation strongly correlates with aggravated hepatocellular necroinjury, upregulated circulating fibrosis serum markers, and increased peripheral monocyte counts. After adjusting for viral load, sex and other disease severity confounders, the crude association between ALT and monocyte abundance was attenuated, demonstrating that ALT acts as a composite systemic biomarker reflecting overall hepatic inflammatory burden rather than an independent driver of monocyte recruitment. Consistent pro-inflammatory cytokine cascades accompanied this ALT-associated injury signature, characterized by downstream signals promoting myeloid cell chemotaxis and tissue inflammatory amplification.

Transcriptomic deconvolution of the GSE84044 liver biopsy cohort further validated elevated intrahepatic monocyte infiltration and predominant M1 macrophage polarization in high-ALT patients, accompanied by dysbalanced adaptive immunity including expanded CD8+ T cells and depleted regulatory T cells (Tregs). GSEA and Spearman correlation verified coordinated activation of the WNT/*β*-catenin signaling axis in ALT-high liver tissues; among all tested pathway genes, APC, MYC, CCND2 and FZD7 exhibited the strongest statistically significant positive correlations with serum ALT (FDR q < 0.001). This signaling signature coincided with upregulated CCR2, a master chemokine receptor mediating monocyte hepatic recruitment.

This study establishes correlative transcriptomic evidence linking WNT/*β*-catenin cascade activation, monocyte infiltration, and unbalanced pro-inflammatory immune microenvironment in chronic HBV liver injury. All observed molecular and cellular associations remain correlative rather than causal, requiring *in vitro* functional assays and *in vivo* animal models to validate regulatory interactions between WNT signaling, cytokine release and macrophage polarization. Collectively, these findings provide preliminary clues for developing CCR2 or WNT pathway-targeted immunomodulatory therapies to mitigate excessive hepatic inflammation and slow fibrotic progression in CHB patients stratified by ALT inflammatory status.

## Data Availability

The original contributions presented in the study are included in the article/Supplementary Material, further inquiries can be directed to the corresponding author.

## Supplementary material

Supplementary Figure 1 Spearman's rank correlation analysis between ALT levels and WNT/β-catenin pathway components. Spearman's rank correlation (ρ) was computed between ALT and each of 17 WNT/β-catenin pathway genes. Benjamini–Hochberg FDR correction was applied across all 16 parallel correlation tests, with FDR-adjusted q < 0.05 as the significance threshold.
