# Supplementary material for: Elevated ALT correlates with severe liver injury, monocyte infiltration, and Pro-inflammatory immune dysregulation in chronic hepatitis B virus infection
Source: Front Med (Lausanne). 2026 Sep 4;13:1865627. doi: 10.3389/fmed.2026.1865627 (PMC13585659; doi:10.3389/fmed.2026.1865627)

Supplementary Figure 1. Spearman's rank correlation analysis between ALT levels and WNT/β-catenin pathway components. Spearman's rank correlation (ρ) was computed between ALT and each of 17 WNT/β-catenin pathway genes. Benjamini–Hochberg FDR correction was applied across all 17 parallel correlation tests, with FDR-adjusted $q < 0.05$ as the significance threshold.

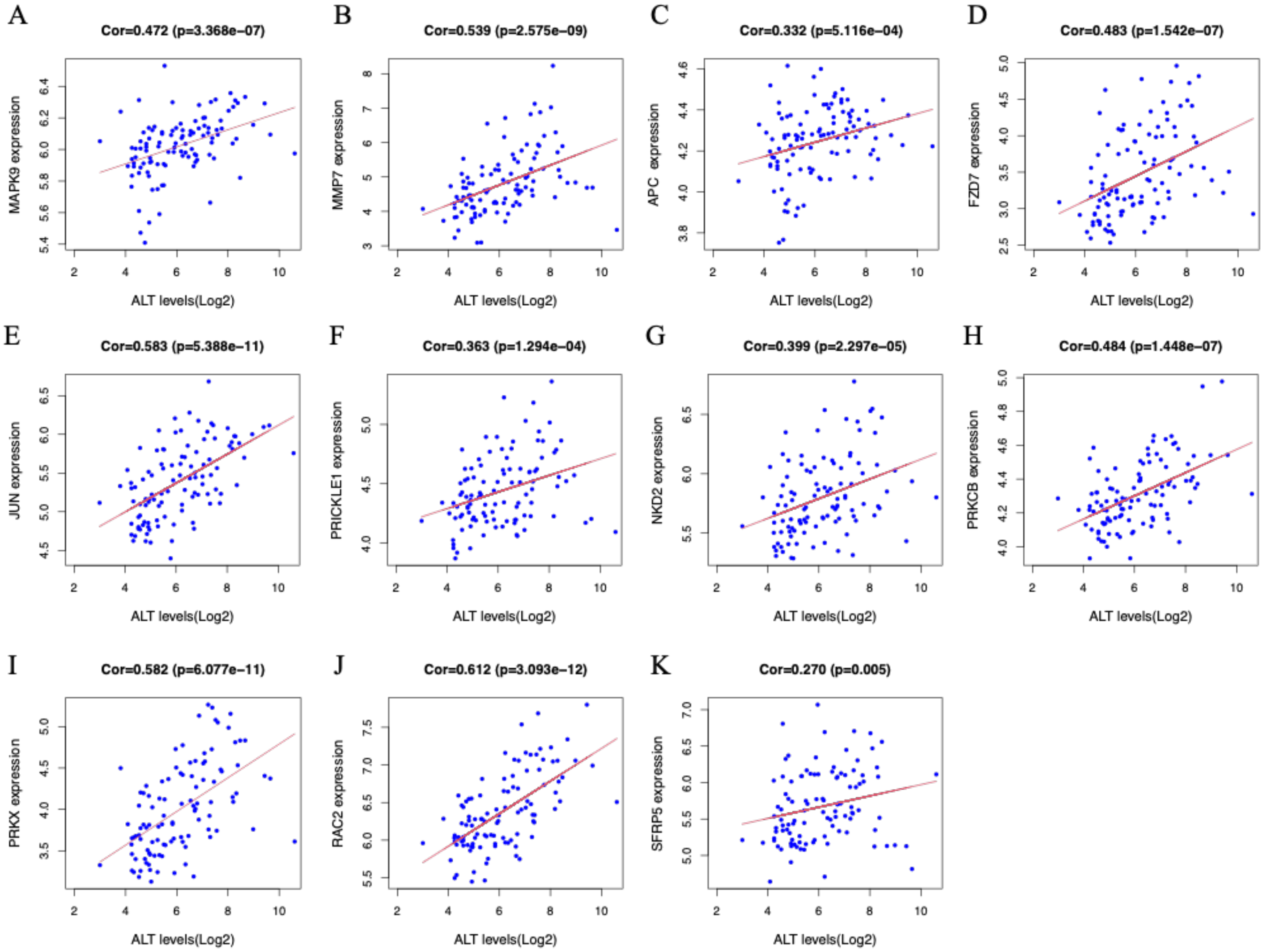

Supplement: Supplementary file 1 [file Image1.pdf]
